# Treatment of Skin Diseases, Small-pox, Syphilis, and Ague in Morocco

**Published:** 1851-09

**Authors:** 


### PATHOLOGY AND PRACTICE OF MEDICINE.

*Treatment of Skin Diseases, Small-pox, Syphilis, and Ague in Morocco.*—We extract from an interesting paper of Dr. Dulac, a French surgeon employed in the embassy to Morocco, the following particulars. The most frequent diseases in Morocco are tænia, the itch, small-pox, syphilis and ague. The *favus vulgaris* is much more common than the *scutiformis*, and the *theubibs* (or doctors) employ the following remedy against it:—A water tortoise is thrown into a large fire, and is there left until it is completely carbonized; it is then taken out and pounded in a mortar. The powder mixed with oil, is every evening smeared on the patient's head, who washes off the ointment in the morning with sea-water. A great many cures are said to be thus obtained. Almost all the Arabs use for the itch an application of sulphur and butter, with which they rub the whole body; in the mountains, however, they prefer a purgative composed of pitch and very rancid butter. In obstinate cases they use an oinment composed of one drachm of arsenious acid to two ounces of butter. Three days' frictions all over the body are said to be sufficient for a cure. Small-pox destroys a frightful number of people, especially in the spring, and as much as one-fourth, or even one-third of the inhabitants of certain localities are carried off. The Moroccese obstinately refuse to be vaccinated, and say that the law of the Prophet is against the practice. The Jews are less averse to vaccination, and Dr. Dulac succeeded in vaccinating several children belonging to Jewish parents, but not one Musselman child was ever brought to him. The *theubibs* say that the small-pox lasts just nine days, and they divide these into three periods. They state that the pustules form durng the first three days, that they fill for the three next, that they empty hemselves and dry up for the three last, and that the patient is out of danger on the tenth day. He is prevented from taking any fish, or herbs, and is given weak tea and chicken broth ; the variations of temperature are avoided, and lemons or vinegar are carefully excluded from the patient's room. Castor-oil, or very rancid butter, is afterwards used as a purgative ; the latter being sometimes mixed with a little tar.

Syphilitic affections are very common in Morocco ; syphilis is called *merd el kebir* (the big disease.) Dr. Dulac saw principally secondary and tertiary affections, which chiefly occupied the anal region. The *theubibs* treat syphilis either with or without mercury. When the metal is not used, the *Haskba* (sarsaparilla) is given, of which the patient is made to *eat* one ounce per diem. He is desired to swallow the whole root, as the doctors do not believe that water can take up its medical properties. The patient avoids tea and coffee, and takes only bread and dried fruit. He continues this for forty days, is then smartly purged, and allowed to resume his usual mode of life. When this treatment does not succeed, and ulceration of the penis or a discharge from the urethra persists, mercury, extinguished in honey, is given. No other mercurial preparation is known. Five grains are given once a week, and a strong dose of castor-oil is administered soon afterwards.

Ague is very common, and though the *theubibs* know the properties of bark, they and the patients avoid its use, owing to its high price ; they substitute for it ground coffee mixed with lemon-juice, and likewise various kinds of bitters. A very celebrated *marabout* in the neighbourhood of the city of Morocco, possesses an infallible specific for ague, but it requires more than common courage to take it. It consists of a slice of bread and butter on which is placed a layer of *pediculi capitis.* Dr. Dulac mentions that this remedy was *proposed* to the wife of a European consul at Tangiers, who could not get rid of her ague, but he does not say whether she *took* it. Dr. Dulac spoke, on the other hand, to several Arabs who had done so, and who spoke very highly of the efficacy of a specific so cheap and so easily procured. When a *theubib* has exhausted all his remedies without any beneficial results, he resorts to religious practices ; he, for instance, writes a few verses of the Koran on a slip of paper, burns it, and makes the patient swallow the residue. Or he writes on a dinner plate what he had put on the paper, washes off the writing from the plate, and desires the sick man to drink the water used for the purpose. Or he, lastly, proceeds like the *mesmerisers* of this country, makes several passes over the head of the patient, and mutters some prayers. As a *dernier resort* the patient undertakes a pilgrimage to some holy and celebrated *marabout*, where he offers up in sacrifice, either a bull, a sheep, a goat, or only a hen, according to his means.—*London Lancet.*

